# Utility of Serial Microbial Cell-free DNA Sequencing for Inpatient and Outpatient Pathogen Surveillance Among Allogeneic Hematopoietic Stem Cell Transplant Recipients

**DOI:** 10.1093/ofid/ofae330

**Published:** 2024-07-03

**Authors:** Monica Fung, Nimish Patel, Catherine DeVoe, Caitlin N Ryan, Staci McAdams, Meenakshi Pamula, Aditya Dwivedi, Justin Teraoka, Matthew Smollin, Srey Sam, Bradley Perkins, Peter Chin-Hong

**Affiliations:** Division of Infectious Diseases, University of California San Francisco, San Francisco, California, USA; Skaggs School of Pharmacy & Pharmaceutical Sciences, University of California San Diego, La Jolla, California, USA; Division of Infectious Diseases, University of California San Francisco, San Francisco, California, USA; Karius, Inc., Redwood City, California, USA; Karius, Inc., Redwood City, California, USA; Division of Infectious Diseases, University of California San Francisco, San Francisco, California, USA; Division of Infectious Diseases, University of California San Francisco, San Francisco, California, USA; Division of Infectious Diseases, University of California San Francisco, San Francisco, California, USA; Karius, Inc., Redwood City, California, USA; Karius, Inc., Redwood City, California, USA; Karius, Inc., Redwood City, California, USA; Division of Infectious Diseases, University of California San Francisco, San Francisco, California, USA

**Keywords:** cytomegalovirus, hematopoietic stem cell transplant, plasma microbial cell-free DNA sequencing, surveillance, molecular diagnostics

## Abstract

**Background:**

This study characterizes the clinical utility and validity of the Karius test (KT), a plasma microbial cell-free DNA sequencing platform, as an infection surveillance tool among hematopoietic stem cell transplant (HCT) recipients, including monitoring for cytomegalovirus (CMV) and detecting infections relative to standard microbiologic testing (SMT).

**Methods:**

A prospective, observational cohort study was performed among adult HCT recipients as inpatients and outpatients. Serial KTs were performed starting with 1 sample within 14 days before HCT, then weekly from 7–63 days posttransplant then monthly from 3–12 months post-HCT. Diagnostic performance of KT versus CMV polymerase chain reaction was evaluated with positive percent agreement and negative percent agreement. Infectious events (<12 months post-HCT) were extracted from medical records. For infectious events without positive SMT, 2 clinicians adjudicated KT results to determine if any detections were a probable cause. Difference in time from KT pathogen detection and infection onset was calculated.

**Results:**

Of the 70 participants, mean age was 49.9 years. For CMV surveillance, positive percent agreement was 100% and negative percent agreement was 90%. There was strong correlation between CMV DNA and KT molecules per microliter (*r*^2^: 0.84, *P* < .001). Of the 32 SMT+/KT+ infectious events, KT identified 26 earlier than SMT (median: −12 days) and an additional 5 diagnostically difficult pathogens identified by KT but not SMT.

**Conclusions:**

KT detected CMV with high accuracy and correlation with quantitative polymerase chain reaction. Among infectious events, KT demonstrated additive clinical utility by detecting pathogens earlier than SMT and those not detected by SMT.

## Teaser

Among stem cell transplant recipients, serial microbial cell-free DNA testing as an infection surveillance tool demonstrated high accuracy for cytomegalovirus monitoring as well as earlier pathogen detections than standard microbiologic testing (SMT) and increased yield when SMT was negative.

## BACKGROUND

Infections are common among recipients of hematopoietic stem cell transplantation (HCT) and a significant source of mortality and health care resources [1–4]. Because of this, improved methods for diagnosing and monitoring of infections among HCT recipients are crucial for patient outcomes [3]. Existing strategies include preemptive monitoring for opportunistic infections, such as serial quantitative polymerase chain reaction (qPCR) testing for cytomegalovirus (CMV) or galactomannan screening for invasive Aspergillosis among patients not on mold-active antifungal prophylaxis. Another approach involves empiric antimicrobials at the onset of clinical signs and symptoms of infection (eg, neutropenic fever, pulmonary nodules) followed by expeditious diagnostic workup to target appropriate therapy.

However, conventional methods to diagnose infections are limited because of low culture yield, difficulty isolating fastidious organisms, laboratory variability in reference ranges (eg, CMV), and the inability to detect all pathogens without requiring a battery of tests [5–9]. The emerging technology of microbial cell-free DNA (mcfDNA) sequencing, such as the Karius test (KT), may overcome some of the known limitations of conventional methods of pathogen detection [10, 11]. For example, among immunocompromised patients with febrile neutropenia in which a causative pathogen is only identified in one third of cases, KT improved the diagnostic yield when compared to standard microbiologic testing (SMT) methods [12–16]. Additionally, KT provides a quantitative measure in molecules per microliter (MPM) that have been previously shown to be correlated with existing laboratory measurements such as international units per milliliter and allow pathogen density to be incorporated into clinical interpretation [17]. Indeed, KT may be an effective pathogen-agnostic surveillance tool to allow for monitoring and earlier detection of infection in immunocompromised patients, yet utility has not yet been assessed prospectively in existing studies [18].

The overall intent of this study was to characterize the clinical utility and validity of the KT as a surveillance tool for infection among HCT recipients as inpatients and outpatients, including monitoring for CMV, potential earlier detection of pathogens relative to SMT, and identification of diagnostically challenging pathogens.

## METHODS

### Study Design and Setting

A prospective, observational cohort trial (ClinicalTrials.gov ID: NCT02804464) was performed among adult HCT patients receiving care at the University of California San Francisco Health between August 2016 and May 2018 to explore the utility of plasma mcfDNA sequencing for detecting infections in this population. Individuals were included in the study if they were age ≥ 18 years, receiving an allogeneic HCT but before the start of their conditioning regimen, and willing to submit to serial blood sample collections in inpatient and outpatient setting.

The specific study objectives were to: (1) assess the diagnostic performance of KT at detecting CMV in the HCT population including correlation between KT MPM and CMV viral load determined by qPCR and (2) determine if surveillance KT could result in earlier detection of clinically relevant pathogens relative to SMT and identify the types of pathogens that could be detected using KT when SMT is negative.

### Patient Consent Statement

Design of study was approved by University of California San Francisco institutional review board (#15-18 026) and written consent was obtained from all participating subjects.

### Karius Testing

KT has the capacity to identify and quantify mcfDNA from >1000 clinically relevant pathogens in plasma [17]. Serial blood samples were collected to perform the KT, starting with 1 sample within 14 days before HCT, then weekly from day +7 to +63, and then monthly from 3 months to 12 months post-HCT. Samples were collected and processed in batches using the 3.11 pipeline and as previously described [17]. Plasma mcfDNA of microorganisms that are determined to be significantly higher than real-time background control specimens were reported and quantified in mcfDNA levels [17]. KT results were not made available to the clinical care team.

### Institutional Infection Prophylaxis and Monitoring Strategies

Allo-HCT recipients are routinely provided antibacterial (fluoroquinolone or third-generation cephalosporin), antifungal (voriconazole or posaconazole), antiviral (acyclovir), and anti-*Pneumocystis* (trimethoprim-sulfamethoxazole) prophylaxis. For CMV D−/R+ and D+ recipients, the CMV prevention strategy used during the study period was preemptive monitoring with weekly CMV viral load and initiation of antiviral therapy at treatment doses at any level above assay detection (137 IU/mL). CMV D−/R− recipients were administered CMV-seronegative blood products. Letermovir was added to institutional formulary and used for CMV prophylaxis of high-risk HCT recipients starting in early 2018. Routine monitoring of other DNA viruses such as human polyomavirus 1 (BKV), human herpesvirus (HHV)-6, and Epstein Barr (EBV) was not performed and was instead sent by primary oncology or infectious diseases at time of clinical concern for infection.

### Data Elements

Data elements that were captured during the course of the study and used in the present analyses included demographics, medical history, medications, laboratory results, infectious events, and vital status within 12 months of HCT. Demographics variables included age, sex, race/ethnicity, height/weight, and calculated body mass index. Variables related to medical history were indication for transplant, allogeneic stem cell source (peripheral/cord blood or bone marrow), donor type (matched related/unrelated or mismatched), conditioning regimen intensity, disease status at time of HCT, and CMV donor/recipient status.

Laboratory values included results for SMT with applicable antimicrobial susceptibilities when an infection was suspected using qPCR testing and KT results. With the exception of KT, SMT including qPCR testing was performed as part of standard of care (SOC) and completed at laboratories affiliated with the institution. The assay used for CMV was COBAS AmpliPrep/COBAS TaqMan CMV test with a range of detection from 137 IU/mL to 9.1 × 10^6^ IU/mL. The qPCR assays (range of detection) used for BKV (100 IU/mL to 2 × 10^7^ IU/mL), EBV (500 IU/mL to 5 × 10^6^ IU/mL), and HHV-6 (1250 IU/mL to 12.5 × 10^6^ IU/mL) were laboratory-developed tests in a Clinical Laboratory Improvement Amendments–approved laboratory.

### Additive Diagnostic Value and KT Pathogen Adjudication

Discharge summaries from all admissions during study follow-up period (up to 12 months) were retrospectively reviewed in subjects’ electronic medical record (EMR) to identify potential or confirmed infectious events. For events with no causative pathogen identified by SMT, 2 infectious diseases clinicians (authors M.F. and C.D.) reviewed pertinent fields documented within the EMR to identify infection onset and adjudicated all preceding KT results to determine if any of the pathogens detected by KT could be potential causes of the infectious event. The pathogens detected by KT were categorized by adjudicators as a probable or unlikely cause of the infectious event. Those that were categorized as probable were considered instances in which the KT provided additive diagnostic value. Onset of infectious event was considered the date of positive SMT (eg, blood culture) confirming diagnosis, first date an individual presented with clinical signs/symptoms associated with an infectious event (eg, fever), or date of procedure diagnosing an infection (eg, chest X-ray). The difference in time from first KT pathogen detection to infection onset was defined as time to infection onset. These cases were subsequently reviewed to evaluate the course of infection including number of positive KTs, MPM distribution, and death within 12 months post-HCT.

### Data Analyses

Descriptive statistics were used to characterize the clinical and demographic features of the study population. Diagnostic performance was evaluated by calculating positive percent agreement (PPA) and negative percent agreement (NPA) [13, 14]. The PPA and NPA calculations were based on the first encounter an individual had a KT and qPCR test drawn within 24 hours of one another. McNemar's test was used to test for marginal homogeneity and determine if agreement between KT and qPCR detections was significant. Test concordance, or overall percent agreement (OPA), was calculated as the total number of tests with dual agreement (KT+/qPCR+ or KT–/qPCR–) divided by the total number of tests performed.

Analyses evaluating the correlation between KT MPM and viral load from qPCR were limited to individuals in which both the KT was positive and there was a measurable viral load from qPCR and both samples were obtained within 1 day of one another at any point during the study period. For individuals with multiple KT+/qPCR+ detections within 1 day of sampling, only the first pair of measurements was used. Pearson's correlation coefficients were computed to assess the correlation between KT MPM and viral load derived from qPCR. These analyses were restricted to the pathogens with a sufficient number of sets of positive test results on both qPCR and KT to reasonably assume the presence of a parametric distribution and make meaningful inferences. Linear regression was used to identify a potential conversion factor between KT MPM and qPCR viral load. Given the nonparametric nature of these data, both KT MPM and qPCR viral load were log-transformed to meet the homoscedasticity assumption. Log_10_[KT MPM] was modeled to predict log_10_[qPCR] with the intercept serving as the potential conversion factor.

Where feasible, stratified analyses were performed to evaluate whether collection samples as an inpatient versus outpatient resulted in effect modification.

## RESULTS

All 70 participants enrolled were included in the analyses. Characteristics of the study population are displayed in Table 1. Overall, the majority (61.4%) of participants were male and the mean age was 49.9 years. The most common indications for HCT were acute myeloid leukemia followed by acute lymphoblastic leukemia, myelodysplastic syndrome, and chronic myelogenous leukemia. For most individuals, the HCT was from a peripheral blood source (81.4%) and from a human leukocyte antigen–matched unrelated donor (62.9%). The CMV donor/recipient (D/R) serostatus was mostly D+/R+ (38.6%) or D−/R+ (32.9%). Two subjects, both D+/R+, received letermovir prophylaxis in the setting of severe graft versus host disease.

**Table 1.** Clinical and Demographic Characteristics of the Study Population

Covariate	Result
Age, mean ± SD	49.9 ± 16.0
Sex	
Male	43 (61.4)
Female	27 (38.6)
Race	
White	43 (61.4)
Unknown/not reported	18 (25.7)
Asian	6 (8.6)
Black	2 (2.9)
More than 1 race	1 (1.4)
Ethnicity	
Not Hispanic or Latino	26 (37.1)
Hispanic or Latino	13 (18.6)
Unknown/not reported	31 (44.3)
Body mass index, median (IQR)	27.6 (24.5–31.1)
Indication for HCT	
Acute myelogenous leukemia	30 (42.9)
Acute lymphoblastic leukemia	9 (12.9)
Myelodysplastic syndrome	8 (11.4)
Chronic myelogenous leukemia	7 (10.0)
Chronic lymphocytic leukemia	2 (2.9)
Non-Hodgkin lymphoma	2 (2.9)
Aplastic anemia	1 (1.4)
Chronic myelomonocytic leukemia	1 (1.4)
Hodgkin lymphoma	1 (1.4)
Other	9 (12.9)
HCT source	
Bone marrow	8 (11.4)
Cord blood	1 (1.4)
Peripheral blood	57 (81.4)
Missing	4 (5.7)
HCT donor type	
HLA-matched related donor	24 (34.3)
HLA-matched unrelated donor	44 (62.9)
HLA-mismatched donor	1 (1.4)
Missing	1 (1.4)
Disease status at time of HCT	
Active disease	13 (18.6)
Chronic phase (CML)	2 (2.9)
First complete remission	37 (52.9)
Second or more complete remission	11 (15.7)
Partial remissions	7 (10.0)
Intensity of conditioning regimen	
Myeloablative	18 (25.7)
Myeloablative/ATG	22 (31.4)
Reduced intensity	6 (8.6)
Reduced intensity/ATG	24 (34.3)
Cytomegalovirus D/R status	
D−/R–	15 (21.4)
D–/R+	23 (32.9)
D+/R–	5 (7.1)
D+/R+	27 (38.6)

All data presented as n (%), mean ± standard deviation, or median (interquartile range, IQR) unless otherwise indicated.

Abbreviations: ATG, antithymocyte globulin; CML, chronic myelogenous leukemia; D, donor; HLA, human leukocyte antigen; R, recipient.

### CMV Surveillance

Over the course of the study, CMV was the most common virus detected by both KT (45) and qPCR (31). The joint relationship between KT and qPCR detections and the calculated PPA/NPA/OPA values counting the first instance when an individual had a KT and/or qPCR test result obtained within 24 hours of one another are displayed in Table 2. The overall PPA and NPA values were 100% and 88.9%, respectively. The *P* value testing for marginal homogeneity demonstrated significant agreement between KT and qPCR detections (<.001). The overall test concordance, where both KT and qPCR were positive or where both were negative, was 89.7%. Among the 7 individuals with a KT+/qPCR– result, there were 6 who had CMV DNA <137 IU/mL measured by qPCR at this sampling time point. Five individuals had a positive qPCR test result the subsequent week. No substantive heterogeneity was observed in PPA/NPA values when the data were stratified by samples collected as an inpatient versus outpatient or CMV donor/recipient status (Table 2).

**Table 2.** Calculated Positive, Negative, and Overall Percent Agreement for Cytomegalovirus Among Individuals’ First Encounter Where Karius Test and Quantitative Polymerase Chain Reaction Tests Were Obtained Within 24 Hours, Stratified by Donor/Recipient and Inpatient/Outpatient Status

Overall		
Calculated PPA: 100%, NPA: 88.9%, OPA: 89.7%		
	qPCR+	qPCR–
KT+	5	7
KT–	0	56
Strata 1: CMV D+/R+		
Calculated PPA: 100%, NPA: 87.0%, OPA: 88.9%		
	qPCR+	qPCR–
KT+	4	3
KT–	0	20
Strata 2: CMV D+/R–		
Calculated PPA: noncomputable, NPA: 100%, OPA: 100%		
	qPCR+	qPCR–
KT+	0	0
KT–	0	6
Strata 3: CMV D–/R+		
Calculated PPA: 100%, NPA: 81.8%, OPA: 82.6%		
	qPCR+	qPCR–
KT+	1	4
KT–	0	18
Strata 4: CMV D−/R–		
Calculated PPA: noncomputable, NPA: 100%, OPA: 100%		
	qPCR+	qPCR–
KT+	0	0
KT-	0	12
Strata 5: Inpatient sample collection		
Calculated PPA: 100%, NPA: 82.6%, OPA: 84.0%		
	qPCR+	qPCR–
KT+	4	8
KT–	0	38
Strata 6: Outpatient sample collection		
Calculated PPA: 100%, NPA: 100%, OPA: 100%		
	qPCR+	qPCR–
KT+	1	0
KT–	0	17

Abbreviations: CMV, cytomegalovirus; D, donor; KT, Karius test, NPA, negative percent agreement; OPA, overall percent agreement; PPA, positive percent agreement; qPCR, quantitative polymerase chain reaction; R, recipient.

**Figure 1.**
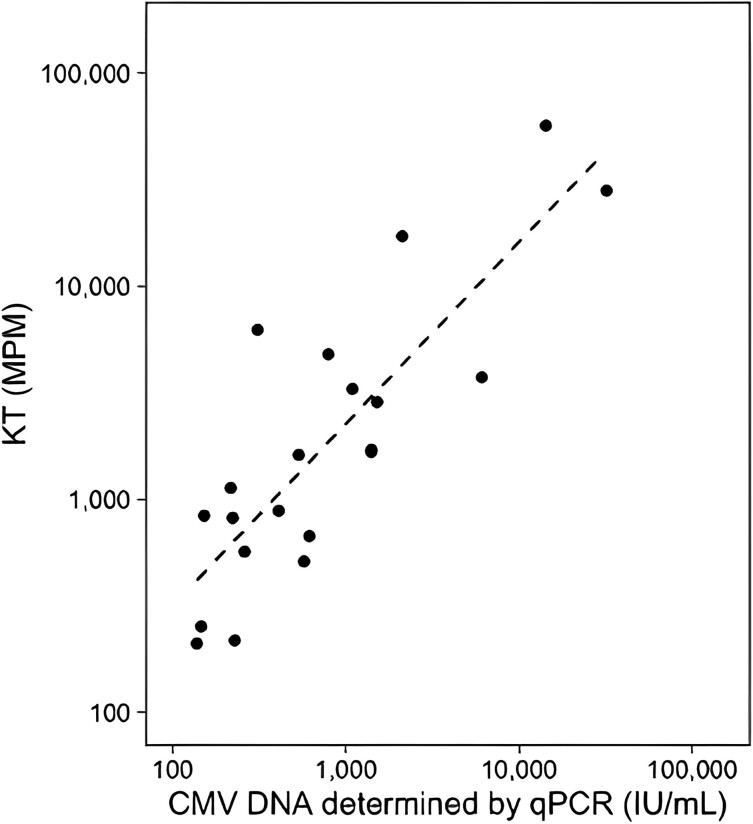
Correlation between plasma CMV DNA and KT MPM values.

The scatter plot of individuals with CMV DNA determined by qPCR and KT MPM obtained within 24 hours at any point during the study period is displayed in Figure 1. There was strong correlation between CMV DNA and KT MPM (*r*^2^: 0.84, *P* < .001). In the linear regression analyses, log_10_[qPCR] was predicted by −0.254 + 0.927 log_10_[MPM].

### Other DNA Viruses

The NPA values for BKV, EBV, and HHV-6 were 92%, 97%, and 96%, respectively. The PPA was 100% for each virus. Overall test concordance was 92%, 97%, and 96% for each of the aforementioned viruses.

### Surveillance Before Infectious Event

**Figure 2.**
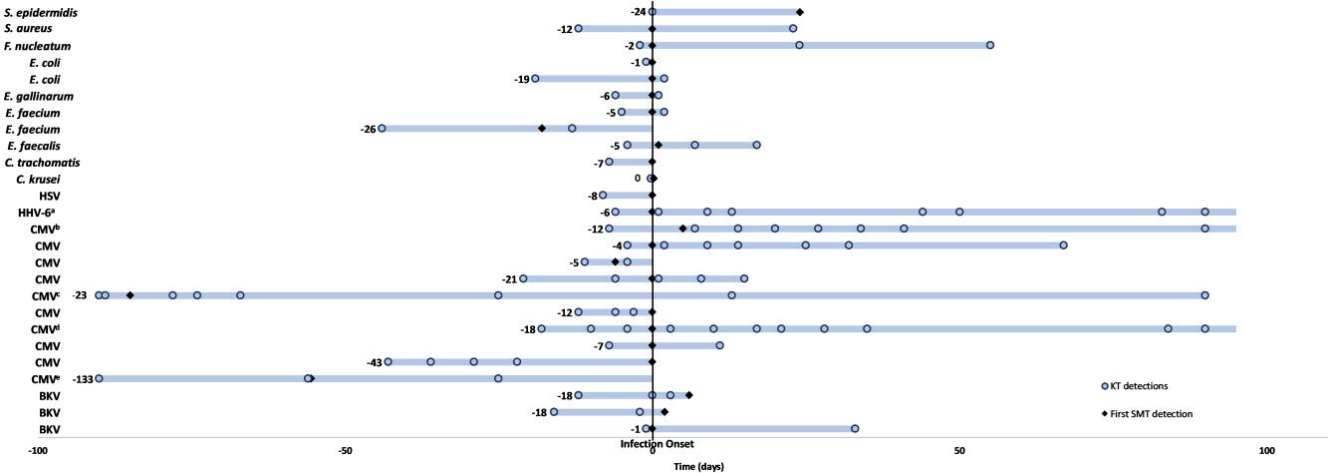
Distribution of pathogens detected earlier by Karius test. Numbers to the left of the bars represent the difference in days between first positive Karius test and first positive value based on standard microbiologic testing (SMT). Infection onset is the date at which individual presented with clinical signs/symptoms associated with infection or had a diagnosing procedure and adjudicated by 2 independent infectious diseases clinicians. ^a^Continued to have positive KT detection on +153 d after infection onset. ^b^Continued to have positive KT detections on +99, + 132, + 162, + 181, and +230 d after infection onset. ^c^Truncated scale on left; first KT detection was at day −108, followed by −102 and −94 d before SMT detection on day −85 before infection onset. ^d^Continued to have positive KT detection on +113 d after infection onset. ^e^Truncated scale on left; first KT detection was at day −189 before SMT detection on day −56 preceding infection onset.

During the 12 months post-HCT, 107 potential or confirmed infectious events occurred in 47 unique participants (Supplementary Table 1). All pathogens detected by KT are displayed in Supplementary Table 2. Among the 32 infectious events with pathogen detections by both KT and SMT (KT+/SMT+), 26 were detected earlier with KT (Figure 2). The most common pathogens were CMV and BKV. The median (interquartile range) time to infection onset after HCT was 20 (13–46) days. The median difference in time to detection between KT and SMT was −12 (−23 to −5) days. There were 11 nonviral infectious events detected by both KT and SMT where KT was earlier by a median (interquartile range) difference of −6 (−19 to −2) days. In nearly half (46%) of these infectious events, the pathogen was detected multiple times by KT before SMT. Among all but 2 of these events with multiple KT detections, the KT MPM increased at least 0.5 log between preceding KTs and the KT immediately before SMT identification.

Of the 6 infectious events in which KT pathogen detection occurred later than SMT, all were detected with the next KT surveillance test immediately following the SMT detection. There were 3 infectious events that occurred when the KT surveillance testing intervals were 30 days apart (Supplementary Table 1).

There were 6 pathogens detected by KT that were adjudicated as a probable cause of 5 infectious events that SMT did not identify (KT+/SMT–) and considered instances in which surveillance KT provided additive diagnostic value (Table 3). Two of these cases were pneumonia (*Bordetella hinzii* and *Mycoplasma hominis*). There was 1 polymicrobial peritonitis/intraperitoneal abscess (*Enterococcus faecium/Escherichia coli*). The median (range) time to infection onset from HCT was 37 (18–155) days. The KT detected pathogens a median (range) of 15 (3–57) days earlier than infection onset. Among these patients, 3 (*B hinzii*, *M hominis*, adenovirus) did not receive effective antimicrobial therapy for their infection, and 2 died within 30 days of being admitted to the hospital for their infection type.

**Table 3.** Disposition of Participants Where Karius Test Provided Additive Diagnostic Value

Case	Pathogen(s)	Infection Type	Number 0f KT Detections Before Infection Onset	KT MPM Range Before Infection Onset	Days From HCT To KT Detection	Days From HCT To Infection Onset	Time To Infection Onset	Number Of Detections After Infection Onset	KT MPM Range After Infection Onset	Antimicrobials^a^	LOS	Days Between Death and Infection Onset
1	*Bordatella hinzii*	Pneumonia	6	52–1100	43	100	57	0	N/A	Before infection onset but after KT detection ciprofloxacin ppx (35) TMP/SMX ppx (14) After infection onset cefepime (10)	11	11
2	*Mycoplasma hominis*	Pneumonia	1	61	15	18	3	0	N/A	Before infection onset but after KT detection TMP/SMX (3) ——– After infection onset: TMP/SMX (160)	29	Alive at 12 mo post-HCT
3	Human mastadenovirus	Ileitis with diarrhea	1	341	140	155	15	0	N/A	After infection onset Acyclovir (2)	2	Alive at 12 mo post-HCT
4	*Escherichia coli* *Enterococcus faecium*	Peritonitis/intraperitoneal abscess	2 3	285–1823 71–439	8	37	29 and 15	0	N/A	Before infection onset but after KT detection: Levofloxacin (1) Cefepime (7) Metronidazole (5) Rifaximin (25) Vancomycin (11) Ciprofloxacin (2) TMP/SMX (15) *———* After infection onset: Ceftriaxone (5) Vancomycin (8) Ciprofloxacin (15)	42 + 15	29
5	HSV-2	Lesion on mons pubis	2	14–922	8	21	13	2	45–95	Before infection onset but after KT detection: Acyclovir ppx (4) —– After infection onset: Acyclovir (25)	47	Alive at 12 mo post-HCT

Abbreviations: HCT, hematopoietic stem cell transplant; HSV-2, herpes simplex virus 2; KT, Karius test; LOS, length of stay; MPM, molecules per microliter; ppx, prophylaxis; TMP/SMX, trimethoprim/sulfamethoxazole.

^a^Bracketed text refers to number of days.

Among 24 cases in which SMT detected probable pathogen and KT was negative (KT–/SMT+), 5 were RNA viruses (metapneumovirus, rhinovirus, influenza, parainfluenza), 9 were *Clostridium difficile* infection, and 5 were bacteremias (*E coli*, *Lactobacillus spp.*, *Mycobacterium fortuitum*, *Staphylococcus epidermidis*, *Streptococcus pyogenes*).

There were 45 infectious events with no pathogen detections by either KT or SMT. The most common infection types were fever (35.6%), rash (8.9%), pneumonia (6.7%), sepsis (6.7%), and central line exit site infection (6.7%).

On an individual level, the overall PPA, NPA, and OPA were calculated for the first infectious episode. The calculated PPA was 12/15 (80.0%). The 3 KT+/SMT– cases were *B hinzii, M hominis*, and human mastadenovirus. The overall NPA was 27/32 (84.3%). The 5 SMT+/KT– cases were *C difficile* toxin assays (n = 2), urine culture with *S epidermidis, S aureus* cellulitis, and 1 respiratory sample with metapneumovirus. The overall OPA was 82.9%.

## DISCUSSION

Prompt identification of pathogens associated with infections in the HCT population is of paramount importance because it may impact time to effective antimicrobial therapy, which is 1 of the strongest predictors of positive clinical outcomes [19]. Surveillance testing is 1 potential tool that could be used to facilitate this. In this cohort of post-HCT patients, KT demonstrated high accuracy and utility at detecting clinically relevant pathogens when used as a surveillance tool. This was evidenced by the high test concordance between KT and CMV qPCR, with strong correlation between CMV DNA in IU/mL and KT MPM. For pathogens detected by both KT and SMT, pathogen detection was earlier using surveillance KT. For infectious events where SMT did not identify any pathogen, KT demonstrated additive diagnostic value as it detected multiple pathogens adjudicated as probable causes of infectious events.

These data have a number of implications for clinical care. Understanding the correlation between CMV DNA and KT MPM will improve clinicians’ understanding of how to interpret CMV detections when using the KT. We identified a conversion between CMV DNA and KT MPM where log_10_[qPCR] = −0.254 + 0.927 log_10_[MPM]. Using this equation, KT MPM of 379 approximates CMV DNA of 137 IU/mL and KT MPM of 3238 is roughly CMV DNA of 1000 IU/mL. The equation is derived from a limited number of samples and requires further validation in larger studies that will likely affect precision.

Use of KT as an adjunctive surveillance tool was able to detect clinically relevant pathogens several days ahead of SMT, especially when there were multiple preceding KT detections and the KT MPM change by more than 0.5 log (Figure 2). Although the median time to detection was 12 days, this may have been longer given testing intervals (monthly from 3–12 months post-HCT). This could enable clinicians to perform enhanced monitoring for signs and symptoms of infection in the days after KT detection and minimize delays in time to appropriate antimicrobial therapy. An additional area that may merit further exploration is the use of KT as a potential antimicrobial stewardship tool to guide the duration of therapy because there were KT detections with declining MPM values after initiation of antimicrobial therapy. Ascending KT MPM values while on antimicrobial therapy could potentially help predict treatment failure or resistance.

Finally, KT can identify pathogens where SMT is not capable and this can potentially affect delivery of care and impact outcomes. Specifically, among the five patients where KT demonstrated additive diagnostic value **(**Table 3), most had infection types that are difficult to obtain a specimen for cultures and susceptibilities (eg, pneumonia and peritonitis). Fastidious organisms (*B hinzii* and *M hominis*) were the causative pathogen in 2 cases. Two patients died within 30 days of being admitted to the hospital. Availability of real-time KT results may have resulted in earlier initiation of antimicrobial therapy and possibly averted a hospital admission, unnecessary antimicrobial therapy, and/or death. Collectively, these findings support the need for a larger study in which KT results are available in real time and focus on meaningful clinical/economic outcomes such as time to antimicrobial therapy, avoidance of hospitalizations, duration of hospitalization if admitted, time to infection resolution, hospital readmissions, and mortality.

There are limitations that should be noted when interpreting these findings. Specifically with regard to CMV results, letermovir received U.S. Food and Drug Administration approval for CMV prophylaxis during the latter part of the study period [20]. Only 2 study subjects received letermovir and neither experienced an adverse CMV-related outcome. As letermovir has become widespread for high-risk HCT recipients, further study into KT MPM must be conducted to determine KT MPM interpretation in the setting of prophylaxis, including identification of patients who could benefit from longer courses (>100 days) of letermovir prophylaxis. Outside of CMV, there was a low number of detections of DNA viruses of interest in the HCT population. Because qPCR for EBV, HHV-6, and BKV was only sent on clinical suspicion (eg, hemorrhagic cystitis), there may be an inherent detection bias that could affect PPA/NPA and should be interpreted in this context. Furthermore, qPCR for BKV, EBV, and HHV-6 were laboratory-developed tests at the institution. Although this could affect the external validity of the findings, it reflects a practice that is likely occurring at other institutions. Finally, some non-KT laboratories performed as part of routine SOC were performed at different laboratories using different assays. This could have affected the precision of the point estimates reported if assays with different reference ranges were being used.

With regard to test performance, KT is inherently limited by DNA-only sequencing, as evidenced by cases of RNA viral infections where SMT alone was positive. There were also 9 cases of *C difficile* not detected by KT and likely a function of the pathogen being isolated to the gastrointestinal tract. Additionally, KT is highly sensitive and may identify mcfDNA from commensal organisms known to be associated with disease but could also represent normal microbiota. Although KT laboratory reports differentiate obligate/opportunistic pathogens that result in disease when detected at any quantity in humans from those that are commensal, it will be imperative to exercise clinical judgment when interpreting KT results and avoid initiating unnecessary antimicrobial therapy.

With regard to study design, this trial was performed at a single site so it is unclear if differences in institutional practices limit the external validity. Infectious events were those that were listed in the discharge summary of the patients’ EMR during the study period. There was no further evaluation of whether the infection listed in the EMR met clear clinical criteria, limiting specificity of infectious events. Although KT was conducted for surveillance, the only surveillance SOC test was CMV PCR, which limits comparative estimates of KT to SMT in terms of timing of pathogen detection. Additionally, samples that underwent the KT were analyzed in batches and were not used to make clinical decisions in real time. This could have influenced whether subsequent SMT was performed to confirm the presence of a potential pathogen and timing/initiation of antimicrobial therapy. As a result, these findings need to be validated in a larger clinical arena where KT detections are available in real time.

Serial KT may be a useful surveillance tool for infection among HCT recipients in the inpatient and outpatient setting. There was strong correlation between KT MPM and quantitative CMV PCR values. KT was able to detect pathogens several days earlier than SMT as well as identify pathogens not detected by SMT. Each of these findings has potential implications for clinical management and patient outcomes. Further study of surveillance KT is warranted to determine its ability to improve timing and yield of pathogen detection in the post-HCT period.

## Supplementary Data

Supplementary materials are available at *Open Forum Infectious Diseases* online. Consisting of data provided by the authors to benefit the reader, the posted materials are not copyedited and are the sole responsibility of the authors, so questions or comments should be addressed to the corresponding author.

## Supplementary Material

ofae330_Supplementary_Data

## Notes

***Acknowledgments*.** The authors acknowledge the contributions of Nicholas Degner, MD, MPH, in the development and execution of the clinical trial.

***Author contributions.*** M.F.: Study design, data collection, clinical review, manuscript writing and revision. N.P.: Manuscript writing and revision. C.D.: Clinical review. C.R.: Data analysis. S.M.: Manuscript revision. M.P.: Data collection. A.D.: Data collection. J.T.: Data collection. M.S.: Manuscript revision. S.S.: Study design, data collection, data analysis. B.P.: Manuscript revision. P.C.H.: Study design, manuscript revision.

***Financial support*.** This work was supported by Karius, Inc. (Redwood City, CA).
