# Supplementary material for: Utility of Serial Microbial Cell-free DNA Sequencing for Inpatient and Outpatient Pathogen Surveillance Among Allogeneic Hematopoietic Stem Cell Transplant Recipients
Source: Open Forum Infect Dis. 2024 Jul 3;11(8):ofae330. doi: 10.1093/ofid/ofae330 (PMC11288372; doi:10.1093/ofid/ofae330)
Supplement: ofae330_Supplementary_Data [file ofae330_supplementary_data.docx]

**Supplemental Table 1**. All Infectious Events and Corresponding Karius Test® and Standard Microbiologic Testing Detections

| **Number of Episodes** | **Clinical Syndrome/Infection Type** | **Karius Test® detection** | **Standard Microbiologic Testing detection** | **Days between infection onset and preceding study visit** |
| --- | --- | --- | --- | --- |
| **Both KT and SMT positive** | | | |  |
| 2 | Bacteremia | *E. coli* | *Escherichia coli* | 4 |
| 1 | Bacteremia | *Enterococcus faecalis* | *Enterococcus faecalis* | 4 |
| 2 | Bacteremia | *Enterococcus faecium* | *Enterococcus faecium* | 5 |
| 1 | Bacteremia | *Enterococcus gallinarum* | *Enterococcus gallinarum* | 6 |
| 1 | Bacteremia | *Fusobacterium nucleatum* | *Fusobacterium nucleatum* | 2 |
| 2 | Bacteremia | *Staphylococcus aureus* | *Staphylococcus aureus* | 6 |
| 1 | Bacteremia | *Staphylococcus epidermidis* | *Staphylococcus epidermidis* | 8 |
| 1 | Bacteremia | *Staphylococcus mitis* | *Streptococcus mitis* | 4 |
| 1 | Candidemia | *Candida krusei* | *Candida krusei* | 6 |
| 1 | Enterocolitis | *C. difficile* | *C. difficile* Toxin | 4 |
| 11 | CMV, viremia or colitis | CMV | CMV | 6 |
| 3 | Hemorrhagic cystitis | BKV | BKV | 2 |
| 1 | HHV-6 viremia | HHV-6B | HHV-6 | 6 |
| 1 | HSV, anogenital | HSV-2 | HSV, type 2 | 1 |
| 1 | HSV, mucositis | Negative | HSV | 1 |
| 1 | Pneumonia | *Escherichia coli* | *Escherichia coli* | 5 |
| 1 | Urogenital | *Chlamydia trachomatis* | *Chlamydia trachomatis* | 7 |
| **KT positive and SMT negative** | | | |  |
| 1 | Enterocolitis | Human mastadenovirus | Not detected | 16 |
| 1 | Pneumonia | *Bordetella hinzii* | Not detected | 7 |
| 1 | Pneumonia | *Mycoplasma hominis* | Not detected | 3 |
| 1 | Peritonitis | *E. coli, E. faecium* | Not detected | 8 |
| 1 | Rash | HSV-2 | Not detected | 6 |
| **KT negative and SMT positive** | | | |  |
| 1 | Bacteremia | Not detected | *Staphylococcus epidermidis* | 13 |
| 1 | Bacteremia | Not detected | *Lactobacillus spp.* | 23 |
| 1 | Bacteremia | Not detected | *Mycobacterium fortuitum* | 4 |
| 1 | Bacteremia | Not detected | *Streptococcus pyogenes* | 6 |
| 1 | Bacteremia | Not detected | *Escherichia coli* | 8 |
| 9 | Enterocolitis | Not detected | *C. difficile* Toxin | 5 |
| 1 | Cellulitis | Not detected | *Staphylococcus aureus* | 25 |
| 1 | Hepatic failure | Not detected | HSV | 16 |
| 1 | Pneumonia | Not detected | Metapneumovirus | 11 |
| 1 | Pneumonia | Not detected | Influenza A | 6 |
| 1 | Pneumonia | Not detected | Rhinovirus | 25 |
| 1 | URTI | Not detected | Rhinovirus | 6 |
| 1 | URTI | Not detected | Parainfluenza | 1 |
| 1 | UTI | Not detected | *Staphylococcus epidermidis* | 3 |
| 2 | UTI | Not detected | *Escherichia coli* | 3 |
| **KT negative and SMT negative** | | | |  |
| 2 | Abdominal fluid collection | Not detected | Not detected | 2 |
| 3 | Central line exit site infection | Not detected | Not detected | 1 |
| 1 | Cholecystitis | Not detected | Not detected | 1 |
| 4 | Enterocolitis | Not detected | Not detected | 7 |
| 5 | Fever, NOS | Not detected | Not detected | 7 |
| 1 | Invasive fungal infection | Not detected | Not detected | 4 |
| 3 | Meningitis | Not detected | Not detected | 3 |
| 11 | Neutropenic Fever | Not detected | Not detected | 4 |
| 3 | Pneumonia | Not detected | Not detected | 8 |
| 1 | Pulmonary nodules | Not detected | Not detected | 10 |
| 1 | Rash | Not detected | Not detected | 2 |
| 3 | Sepsis, NOS | Not detected | Not detected | 9 |
| 1 | Sinusitis | Not detected | Not detected | 18 |
| 1 | Supraglottitis | Not detected | Not detected | 4 |
| 1 | URTI | Not detected | Not detected | 19 |
| 1 | UTI | Not detected | Not detected | 6 |

*Instances where there was more than one episode, the value represents the median time between KT detection and Abbreviations – BKV: human polyoma virus 1, CMV: cytomegalovirus, HSV: herpes simplex virus, HHV: human herpesvirus, KT: Karius Test®, NOS: not otherwise specified, SMT: standard microbiologic testing, URTI: upper respiratory tract infection, UTI: urinary tract infection.

**Supplemental Table 2.** All Pathogens Detected by Karius Test®

| **Pathogen** |
| --- |
| *Abiotrophia defectiva* |
| *Acidaminococcus intestini* |
| *Acinetobacter baumanii* |
| *Actinomyces graevenitzii* |
| *Actinomyces oris* |
| *Actinomyces odontolyticus* |
| *Acintomyces viscosus* |
| Adeno-associated dependoparvovirus A |
| *Aggregatibacter segnis* |
| *Alloiococcus otitis* |
| *Aspergillus flavus* |
| *Aspergillus sydowii* |
| *Aspergillus tubingensis* |
| *Bacillus cereus* |
| *Bacillus coagulans* |
| *Bacillus licheniformis* |
| *Bacillus paralicheniformis* |
| *Bacillus thuringiensis* |
| *Bacteroides fragilis* |
| *Bacteroides ovatus* |
| *Bacteroides stercoris* |
| *Bacteroides thetaiotaomicron* |
| *Bacteroides uniformis* |
| *Bacteroides vulgatus* |
| **BK polyomavirus** |
| ***Bordatella hinzii*** |
| *Candida parapsilosis* |
| *Candida tropicalis* |
| ***Candida krusei*** |
| *Campylobacter concisus* |
| *Campylobacter showae* |
| *Capnocytophaga gingivalis* |
| *Capnocytophaga sputagena* |
| *Chryseobacterium indologenes* |
| *Citrobacter freundii* |
| *Citrobacter koseri* |
| ***Chlamydia trachomatis*** |
| *Clostridium clostridioforme* |
| ***Clostrioides difficile*** |
| *Clostridium innocuum* |
| *Corynebacterium afermentans* |
| *Corynebacterium aurimucosum* |
| *Corynebacterium matruchotii* |
| *Corynebacterium minutussimum* |
| *Corynebacterium propinquum* |
| *Corynebacterium ureicelerivorans* |
| *Cronobacter sakazakii* |
| *Cyberlindnera fabianii* |
| *Cyberlindnera jadinii* |
| *Delftia acidovorans* |
| *Dermabacter hominis* |
| *Dermacoccus nishinomiyaensis* |
| *Diaporthe longicolla* |
| *Dolosigranulum pigrum* |
| *Enterobacter cloacae* complex |
| ***Enterococcus faecalis*** |
| ***Enterococcus faecium*** |
| ***Enterococcus gallinarum*** |
| *Enterocytozoon bieneusi* |
| *Erwinia billingiae* |
| *Erwinia gerundensis* |
| ***E. coli*** |
| *Finegoldia magna* |
| *Fusarium oxysporum* |
| ***Fusobacterium nucleatum*** |
| *Fusobacterium peridonticum* |
| *Gardnerella vaginalis* |
| *Gemella haemolysans* |
| *Gemella morbillorum* |
| *Granulicatella adiacens* |
| *Granulicatella elegans* |
| *Haemophilus influenzae* |
| *Haemophilus parainfluenzae* |
| *Helicobacter pylori* |
| **Human herpesvirus 1** |
| **Human herpesvirus 2** |
| Human herpesvirus 4 |
| **Human herpesvirus 5 (Cytomegalovirus)** |
| **Human herpesvirus 6B** |
| Human herpesvirus 7 |
| Human herpesvirus 8 |
| **Human mastadenovirus A** |
| Human mastadenovirus C |
| Human mastadenovirus D |
| Human polyomavirus 6 |
| Human polyomavirus 7 |
| JC polyomavirus |
| *Klebsiella michiganensis* |
| *Klebsiella pneumoniae* |
| *Klebsiella variicola* |
| *Kocuria rhizophila* |
| *Lactobacillus acidophilus* |
| *Lactobacillus fermentum* |
| *Lactobacillus gasseri* |
| *Lactobacillus hamnosus* |
| *Lactobacillus pontis* |
| *Lactobacillus rhamnosus* |
| *Leptotrichia wadei* |
| *Leptotrichia buccalis* |
| *Macrococcus caseolyticus* |
| *Malassezia furfur* |
| *Malassezia globosa* |
| *Micrococcus lylae* |
| *Moraxella nonliquefaciens* |
| *Morococcus cerebrosus* |
| *Mycobacterium chelonae* |
| ***Mycoplasma hominis*** |
| *Neisseria elongata* |
| *Neisseria flavescens* |
| *Neisseria mucosa* |
| *Neisseria sicca* |
| *Ophiostoma piceae* |
| *Parabacteroides distasonis* |
| *Peptoniphilus harei* |
| *Peptostreptococcus stomatis* |
| *Pichia kudriavzevii* |
| *Pneumocystits jirovecii* |
| *Prevotella bivia* |
| *Prevotella loeschii* |
| *Prevotella melaninogenica* |
| *Prevotella nigrescens* |
| *Propionibacterium namnetense* |
| *Pseudomonas fluorescens* |
| *Raoultella ornithinolytica* |
| *Rothia aeria* |
| *Rothia dentocariosa* |
| *Rothia mucilaginosa* |
| *Saccharomyces cerevisiae* |
| *Scedosporium apiospermum* |
| ***Staphylococcus aureus*** |
| *Staphylococcus capitis* |
| ***Staphylococcus epidermidis*** |
| *Staphylococcus gallinarum* |
| *Staphylococcus haemolyticus* |
| *Staphylococcus hominis* |
| *Staphylococcus pettenkoferi* |
| *Staphylococcus succinis* |
| *Staphylococcus warneri* |
| *Streptococcus agalactiae* |
| *Streptococcus gordonii* |
| *Streptococcus infantis* |
| ***Streptococcus mitis*** |
| *Streptococcus oralis* |
| *Streptococcus parasanguinis* |
| *Streptococcus pneumoniae* |
| *Streptococcus pseudopneumoniae* |
| *Streptococcus salivarius* |
| *Streptococcus sanguinis* |
| *Streptococcus thermophilus* |
| Torque tenovirus |
| Torque tenovirus 15 |
| Torque tenovirus 16 |
| Torque tenovirus 19 |
| Torque tenovirus 28 |
| *Ureaplasma parvum* |
| *Ustilago hordei* |
| *Veillonella dispar* |
| *Veillonella parvula* |

*Bolded items are Karius Test® detections associated with an infectious event
